# Supplementary material for: “Must you make an app?” A qualitative exploration of socio-technical challenges and opportunities for designing digital maternal and child health solutions in Soweto, South Africa
Source: PLOS Glob Public Health. 2022 Dec 5;2(12):e0001280. doi: 10.1371/journal.pgph.0001280 (PMC10021787; doi:10.1371/journal.pgph.0001280)
Supplement: S2 File — (DOCX) [file pgph.0001280.s002.docx]

| **KII NUMBER:** | **DATE (DD/MMM/YY):** | **START TIME:** | **END TIME:** | **FACILITATOR INITIALS:** |
| --- | --- | --- | --- | --- |

**[Read to participants]**

We are very happy that you took the time to meet with us today.

We are excited to hear your thoughts and opinions about the challenges and problems faced looking after your health and that of your child. By understanding your needs and the needs of other community members like you, we hope to work towards a solution that is useful to you.

I have already read through the consent form with you, which describe this project in detail and you have given us permission to speak with you. As a reminder, you are not required to answer all of our questions, and you may choose to skip questions. As a reminder, we will be audio recording our telephonic conversation today.

The discussion will last approximately 45 minutes. You are the expert and so we are really eager to hear what you have to say. There are no “right” answers – just your own experience and thoughts.

Do you have any questions before we begin the discussion?

***Note to interviewers: Turn on digital recorder.***

I am (FACILITATOR NAME) interviewing (KII ID#) on [DATE] [START TIME]

**Theme 1: Demographics**

- Ages of children in the household (EDD for pregnancy)
- Anyone else in the household which include other caregivers in the household
- Devices and internet access

**Theme 2: Defining the problems**

- Where do you go for help concerning the health of your children?  Explore all possibilities
- How have you used your phone concerning maternal and child health?
- Do you ever use WhatsApp or other social media to look for information or ask people for advice? Tell me more. Can you show me a recent conversation?
- Do you use any maternal and child health apps?  Which ones?
- Are you aware of any campaigns or projects around maternal and child health?
- If no – ask about specific campaigns (national and local, Mom Connect)
- What works about these campaigns?
- What would make the campaigns work better for you?
- If you could create and plan your campaign or project to help mothers, what would

it look like, what would it do, what need would it address?

- What about your friends and neighbours that might not have phones, or live in areas that don’t have a network? Can you talk about what their experience might be like, and what works for them?

**Theme 3: Perception of and challenges around existing campaigns and interventions**

- MomConnect
- NGO interventions
- Messages for Mothers
- Road to Health booklet
- Maternity Case Book
- ECD programs?
- Other resources?
- Customizable interfaces? Use of PHI, apps for MCH?
- Feedback from parents that don’t have phones or smartphones (and therefore they find it is harder to communicate)

**Theme 4: How to do research?**

- What are the most pressing needs for mothers, fathers, caregivers around their health and that of their babies/children in Soweto?
- What solutions/products would you wish you had access to so as to improve your and your baby/child’s health?
- What research/study should we do in Soweto to improve health?
- Any other advice you can give us?

Thank you for helping us understand more about the types of services and programs that can help parents/caregivers like you. The details you have shared here today are very valuable. They will help us as we plan and think about how we might be able to work with you to solve some of these problems.

Thank you for your participation.

***Note to interviewers: Note the time the conversation is concluded. Turn off the digital recorder.***
