# Supplementary material for: “Must you make an app?” A qualitative exploration of socio-technical challenges and opportunities for designing digital maternal and child health solutions in Soweto, South Africa
Source: PLOS Glob Public Health. 2022 Dec 5;2(12):e0001280. doi: 10.1371/journal.pgph.0001280 (PMC10021787; doi:10.1371/journal.pgph.0001280)
Supplement: S4 File — (DOCX) [file pgph.0001280.s004.docx]

**AUTHOR REFLEXIVITY STATEMENT**

**“Must you make an app?” A qualitative exploration of socio-technical challenges and opportunities for designing digital maternal and child health solutions in Soweto, South Africa**

Sonja Klingberg, Molebogeng Motlhatlhedi, Gugulethu Mabena, Tebogo Mooki, Nervo Verdezoto, Melissa Densmore & Shane A Norris

This article is the result of interdisciplinary and international collaboration. The research was undertaken in South Africa, with British funding for the study, and South African, Welsh and Finnish funding for some authors’ work. While all authors have been involved in research in South Africa in various capacities, they do so from different professional, disciplinary, cultural and personal perspectives. The data collection and analysis were carried out by the more junior South Africa-based authors, while the study was designed by the more senior authors MD (South Africa-based), NV (UK-based) and SAN (South Africa-based) with international funding.

The lead author, SK, is a White Finnish woman who has lived and worked in Johannesburg, South Africa for several years with postdoctoral funding from a private Finnish research foundation (Kone Foundation). She has previous experience of conducting qualitative health research in Soweto and many other settings. In Soweto and South Africa more generally, she considers herself an obvious outsider (1), but tends to find that while trust may be unrealistic to expect in once-off interviews, good rapport is achievable through respectful and friendly interactions with participants. SK avoids making analytic claims based on interpretations about culture or attempting to provide ‘insider’ perspectives in other ways. She tries to interrogate her assumptions as much as possible through practising reflexivity (2) and by working and discussing together with local colleagues at all stages of research. SK approaches qualitative health research from a mixed social science background combined with intervention-focused public health research experience, which involves a tension of trying to both understand and change aspects of people’s realities. Some of these tensions are captured in the article’s reflections about ethics.

GM, MM and TM are Black South African women, and work in project coordinator (GM & MM) and research assistant (TM) roles on various projects at Wits University’s Developmental Pathways for Health Research Unit in Soweto. GM and MM have many years of qualitative research experience, and while TM is relatively new to qualitative health research, she has the most specific local knowledge as a resident of Soweto. These three authors have the most relevant linguistic and cultural insights out of the team, and can thus interact with participants and build rapport with more ease. GM and MM conducted interviews, while TM carried out participant recruitment. All three played a key role in analysing data and discussing interpretations with SK, e.g. highlighting the relevance of multiple forms of coping and observing differences between participant groups.

It is important to note that there were some existing relationships between GM, MM and TM and most of the research participants. The community health workers recruited into the study knew GM and MM through an intervention trial they are all working in, and TM used to work as a community health worker in the trial herself. Most interviews (7/8) with community health workers were thus carried out by SK, who does not have existing relationships with any of the participants. The recent mothers in the study will also have been familiar with GM, MM and TM from other research activities, but that familiarity as research participants (as opposed to colleagues) was not considered a barrier for being interviewed by GM and MM.

GM, MM, TM and SK are all new to digital health and co-design of technological solutions, and this may have both helped and hindered their role in conducting interviews with participants and analysing the data. On the one hand, they were able to avoid technical jargon and approach the interviews with relative openness due to limited pre-existing knowledge about the topics of interest. One the other hand, they likely missed some potentially valuable paths of inquiry through not probing at points where interviewers with more thematic expertise might have identified novel insights from participants.

The remaining three authors were not involved in data collection activities, but designed the study as well as contributing in the following ways:

- NV is an Ecuadorian computer science researcher who is based in Wales but has been involved in South Africa-based research for several years, among many other international research projects. He contributed considerably to the analysis and interpretations from the perspective of international audiences, and with specific digital health expertise and research experience from multiple low-income settings.
- MD is an American computer science researcher who has been based in Cape Town, South Africa for several years. She has experience of qualitative research and co-design processes in many African settings, which informed her input to the analysis and interpretations.
- SAN is the director of the Developmental Pathways for Health Research Unit at Wits University. He is a South African research professor, and the local principal investigator of the CoMaCH study in Soweto with over 20 years of health research experience in Soweto. SAN’s contributions to the article stem from locally informed public health and health policy expertise.

While the CoMaCH study and team have high-income country funding, a majority of the research team members are based in South Africa, and can be seen as writing from both local and international perspectives, but predominantly for a foreign gaze (3). Realistically, Anglophone academic culture and publishing conventions (even open access publishing) influence the degree to which the resulting article is practically accessible to wider audiences or truly local, Sowetan audiences. The research team therefore intends to carry out dissemination activities more directly through community engagement and co-design processes rather than expecting academic writing to easily reach participating communities.

References

1. Berger R. Now I see it, now I don’t: researcher’s position and reflexivity in qualitative research: Qual Res [Internet]. 2013 Jan 3 [cited 2022 Jul 5];15(2):219–34. Available from: https://journals.sagepub.com/doi/10.1177/1468794112468475

2. Subramani S. Practising reflexivity: Ethics, methodology and theory construction. Methodol Innov [Internet]. 2019 May 10 [cited 2020 Jan 15];12(2):205979911986327. Available from: http://journals.sagepub.com/doi/10.1177/2059799119863276

3. Abimbola S. The foreign gaze: Authorship in academic global health. BMJ Glob Heal [Internet]. 2019 Oct 1 [cited 2019 Nov 21];4(5):e002068. Available from: http://dx.doi.org/10.1136/bmjgh-2019-002068
