# Supplementary material for: “Must you make an app?” A qualitative exploration of socio-technical challenges and opportunities for designing digital maternal and child health solutions in Soweto, South Africa
Source: PLOS Glob Public Health. 2022 Dec 5;2(12):e0001280. doi: 10.1371/journal.pgph.0001280 (PMC10021787; doi:10.1371/journal.pgph.0001280)
Supplement: Attachment — Submitted filename: PLOS GPH revision response to reviewers.docx [file pgph.0001280.s005.docx]

**Re: Revision of manuscript PGPH-D-22-01244R1**

**Detailed response to reviewers**

| **Reviewer comment** | **Response** |
| --- | --- |
| Reviewer #1: I recommend that the article be accepted. It is a well written article which addresses current and relevant issues related to the use of digital health to improve access to health information and health care in low and middle income countries. Particularly important is that it explores these issues from the user's perspective. | Thank you very much for this encouraging feedback! |
| Reviewer #1: A clear problem statement and rationale for the study is provided, and the methods are well described. However more could be said about the ethics of doing in-person interviews during the COVID-19 pandemic and precautions taken to protect participants and research staff. | Thank you for this suggestion, we have added the following details under the Materials and methods (new text in **bold**).  “As the research unit is situated on a hospital campus, and has the capacity to carry out research following COVID-19 protocols **(e.g. symptom screening)**, these interviews were conducted in-person **in line with guidance and approvals from the University. All research staff and participants wore masks at all times, in accordance with South African COVID-19 regulations at the time. The interviews were carried out in private rooms with open windows and sufficient space for physical distancing.**”  We have not specifically commented on the ethics of doing non-urgent research in a pandemic as we feel such important reflections would require more space than we can afford in this manuscript. We hope that detailing the precautions taken, in combination with having ethical approval from the University, is sufficient to demonstrate that we have minimised controllable risks – including by timing the interviews during a period of relatively low COVID-19 incidence, in between South Africa’s distinct waves of infections. |
| Reviewer #1: The data analysis and results are well presented, and support the discussion and conclusions. | Thank you! |
| Reviewer #1: In reporting and discussing the study findings, it would have been useful to have more sociodemographic information on the participants e.g. education levels, employment. They also appeared to have been participants or community health workers in a related research project. Thus some discussion on how their perspectives may differ from women in other settings such as rural areas in South Africa, or may have been influenced by their proximity to ongoing research on these topic is needed. | We agree. We have added the following to the Discussion section on study limitations to address this despite not having further data to include (new text in **bold**).  **“****Furthermore, the analysis could have benefitted from more contextual and sociodemographic data. Such data could have provided further nuance but were not collected as the study was not designed for sociodemographic representativeness or sub-group analyses."**  Due to limited space in this manuscript, we have not added extensive discussion points about potential differences between urban and rural settings but other components of the CoMaCH project capture this through comparisons between Soweto and other, including rural, research sites. We have added the following (new text in **bold**):  "The participation and inclusion of men**, as well as comparisons between urban and rural research sites,** have also been discussed more thoroughly in later phases of the CoMaCH research and co-design process (17).”  In addition, we have provided some reflections on the existing working relationships and attempts to mitigate related issues in the Author Reflexivity Statement (S4). |
| Reviewer #1: A recent systematic review should on the use of digital health technologies in SA during COVID-19 would be good to refer to in the intro and discussion to reflect on whether this study confirms or differs in its findings:  Mbunge E, Batani J, Gaobotse G, Muchemwa B. Virtual healthcare services and digital health technologies deployed during coronavirus disease 2019 (COVID-19) pandemic in South Africa: a systematic review. Global Health Journal. 2022 Mar 5. | Thank you for this recommendation. We had already cited this paper but agree that it is useful and relevant for other parts of the intro and discussion too, and have added the following (new text in **bold**).  Introduction:  “Existing uses in South Africa and other African countries include reminders about antenatal visits, pregnancy registration, child health information, supporting health behaviours through mobile messages**, and technologies specifically adopted in response to the coronavirus disease 2019 (COVID-19) pandemic and its impact on health care services (Mbunge et al. 2022)**.”  Discussion:  “These findings echo recognised impediments to trust in, **and utilisation of,** digital health ~~systems~~ **technologies**, including cost, **unequal access,** defective technology, poor information quality and inadequate publicity hindering optimal use of already existing digital health solutions (43 **+ Mbunge et al. 2022**).” |
| Reviewer #2: Congratulations to the authors on this very interesting manuscript that is timely and interesting. The methods, results and discussion flow logically and make sense. The work is important and I am happy to see the in-situ research on the intersection of maternal health and ICT. This is the first review manuscript that I have ever recommended be accepted without modifications. | Thank you very much for the encouraging feedback! |
| Editor: Please review your reference list to ensure that it is complete and correct. If you have cited papers that have been retracted, please include the rationale for doing so in the manuscript text, or remove these references and replace them with relevant current references. Any changes to the reference list should be mentioned in the rebuttal letter that accompanies your revised manuscript. If you need to cite a retracted article, indicate the article’s retracted status in the References list and also include a citation and full reference for the retraction notice. | To the best of our knowledge, we have not cited any retracted papers, and have screened all cited papers via the Retraction Watch Database.  We have provided more specific sources for the philosophical stance of the study and changed some of the references to reflect this:  Removed Gareth Wiltshire & Noora Ronkainen (2021) A realist approach to thematic analysis: making sense of qualitative data through experiential, inferential and dispositional themes, Journal of Critical Realism, 20:2, 159-180, DOI: 10.1080/14767430.2021.1894909  Added Noora J. Ronkainen & Gareth Wiltshire (2021) Rethinking validity in qualitative sport and exercise psychology research: a realist perspective, International Journal of Sport and Exercise Psychology, 19:1, 13-28, DOI: 10.1080/1612197X.2019.1637363  Amber J. Fletcher (2017) Applying critical realism in qualitative research: methodology meets method, International Journal of Social Research Methodology, 20:2, 181-194, DOI: 10.1080/13645579.2016.1144401 |
